# Health-related quality of life in Iranian women with polycystic ovary syndrome: a qualitative study

**DOI:** 10.1186/s12905-015-0272-4

**Published:** 2015-11-30

**Authors:** Seyed Abdolvahab Taghavi, Fatemeh Bazarganipour, Siobhan Hugh-Jones, Nazafarin Hosseini

**Affiliations:** Mother & Child Welfare Research Center, Hormozgan University of Medical Sciences, Bandar Abbas, Iran; Health Psychology, School of Psychology, University of Leeds, Leeds, UK; Social Determinants of Health Research Center, Yasuj University of Medical Sciences, Yasuj, Iran

**Keywords:** Polycystic ovarian syndrome, Quality of life, Qualitative research

## Abstract

**Background:**

Polycystic ovary syndrome (PCOS) is associated with a range of challenging symptoms which impact patient’s lives. Iranian women with PCOS are likely to face a number of unique difficulties given particular societal and cultural norms for women. Understanding health-related quality of life (HRQoL) from a patients’perspective is critical to developing the appropriate support interventions. The present study aimed to generate an in-depthunderstanding of HRQoL Iranian women with PCOS.

**Methods:**

Twenty Iranian women were interviewed and data was subjected to thematic analysis.

**Results:**

Women reported substantial effects of PCOS on their quality of life, Themes generated from the data related to sexual - physical problems (An unsexualised self: loss, change and pain; and Being pained and painful); exposure and nvasion: the rejecting and invading social world (Concealing and Avoiding and Public property: public scrutiny), diminished self and diminished life (Infertile as inferior and Exhausted mind andbody) respectively.

**Conclusion:**

PCOS is a physical - sexual, psychological and social syndrome; therefore, it is necessary to taking a more holistic approach to patient care beyond treating physical symptoms.

## Background

Polycystic ovary syndrome (PCOS) is the most common endocrine disorder in women of reproductive age. It is estimated that 5 to 10 % of women suffer from the disease [1]. The symptoms typically associated with PCOS include: amenorrhea, oligomenorrhea, hirsutism, obesity, infertility, anovulation and acne. The impact of this condition on patients’ lives and psychological health is well recognized. Post diagnosis, patients with PCOS face multiple tests and hospital appointments, with associated anxiety. Studies indicate diminished health related quality of life (HRQoL), marital and social difficulties, depression and suicidal ideation among patients with PCOS [2, 3]. Poor QoL is likely to be a risk factor for other poor outcomes. For example, in one study, 14 % of women suffering from PCOS reported suicidal ideation [4]. This figure is similar to that reported by patients suffering from other chronic medical conditions and much higher than that reported by the general population. Culture and society can shape what an illness means to an individual. Iranian women typically take up prominent roles in managing family life, diagnosis of, and its subsequent treatment regime, is likely to have significant and unique effects on the on their HRQoL.

Although HRQoL is a subjective perception of well-being, it has been argued that one’s perception may be influenced by cultural and ethnic factors such as social norms, values and beliefs [5]. For example, it is forbidden for a menstruating woman to perform many religious activates, like prayer; therefore, prolonged bleeding disrupts household patterns in such a way that family and community members may become aware of a woman’s situation if her period persists for more than the expected number of days [6, 7]. Menstrual irregularities may also have adverse consequences for women’s intimate relations and for other aspects of their reproductive and general health. For example, in Islam, Judaism and Zoroastrianism, a man is forbidden to have intercourse with his wife during her menses. Whilst PCOS is well researched from a medical perspective, there is relatively little work on patient experience and perspective. Understanding HRQoL from a patients’ perspective is critical to developing the appropriate interventions for the improvement or maintenance of HRQoL in patients with PCOS. With a view to developing effective and support interventions to women diagnosed with PCOS, the present study aimed to explore the effects PCOS has on the physical, social, and psychological and emotional aspects of the lives of Iranian women.

## Methods

### Study design

This was a qualitative study to explore the various effects that PCOS has on the physical, social, and psychological/emotional aspects of the lives of Iranian women.

### Ethical considerations

The ethics committee of the Yasuj Medical University approved the study. All participants give informed consent to publish the information contained in the manuscript and the participant details included in Table 1. Pseudonyms have been used to protect the women’s anonymity.

**Table 1:** Participant demographics

Participants	Age	Educated	Duration of diagnosed polycystic ovary syndrome	Duration of marriage	Occupation	Reproductive status	Menstruation	BMI	Acne score	Hirsutism score
1	27	M.Sc	9	14	housewife	Primary infertility (1.5 y)	Oligomenorrhea	29	8	4
2	23	M.Sc	10	14	Employed	Primary infertility (1 y)	Normal	24	30	16
3	24	Diploma	8	12	housewife	Primary infertility (3 y)	Oligomenorrhea	27	10	8
4	22	Diploma	4	8	housewife	Primary infertility(1.5 y)	Normal	19	16	4
5	27	M.Sc	12	18	housewife	Primary infertility (6 month)	Oligomenorrhea	24	7	7
6	22	Under diploma	9	8	housewife	Primary infertility (6 month)	Amenorrhea	23	16	8
7	27	B.S	11	12	Employed	Primary infertility (1 year)	Oligomenorrhea	31	0	9
						Secondary infertility(4 month)				
8	22	Under diploma	3	4	housewife	Primary infertility(3 year)	Normal	33	3	9
9	30	Under diploma	4	2	housewife	Primary infertility (1.5 year)	Oligomenoreha	25	8	10
10	25	Diploma	10	12	housewife	Primary infertility (2 year)	Amenorrhea	25	16	3
11	25	Diploma	3	13	housewife	Primary infertility (1 year)	Normal	20	21	9
12	29	Diploma	1	1	housewife	Primary infertility (1 year)	Normal	27	7	3
13	28	Diploma	2	2	housewife	Primary infertility (1 year)	Oligomenorreha	22	8	0
14	25	B.S	10	12	housewife	Primary infertility (1.5 year)	Amenorrhea	25	24	15
15	23	Diploma	3	3	housewife	Primary infertility (2 year)	Normal	25	10	9
16	28	B.S	5	8	Employed	Primary infertility (2 year)	Oligomenorehea	27	9	12
17	34	B.S	10	15	housewife	Primary infertility (8 year)	Amenorrhea	26	17	13
18	21	Diploma	3	3	employed	Primary infertility (2 year)	Normal	23	7	4
19	27	Diploma	4	5	housewife	Primary infertility (4 year)	Oligomenorhea	22	7	9
20	32	Under diploma	10	12	housewife	Primary infertility (9 year)	Oligomenorhea	27	9	5

### Recruitment

Patients diagnosed with PCOS, and who were attending outpatient gynecology clinics in Yasuj and Kashan (Iran), were invited to participate in the study. Before their clinic appointments, patients interested in taking part were screened (through a brief face-to-face consultation) to check if they met the following inclusion criteria: being Iranian and between 15 and 40 years of age; married; not having non-classic adrenal hyperplasia, thyroid dysfunction and hyperprolactinemia; capacity to take part in an interview; no history of psychiatric diagnoses or using psychiatric medications including antidepressants; not taking prescribed medication (except allergy and occasional pain medications) for at least three months before the study; having two of the following Rotterdam diagnostic criteria: (i) polycystic ovaries visualized on ultrasound scan (presence of 12 follicles or more in one or both ovaries and/or increased ovarian volume i.e., >10 ml); (ii) clinical signs of hyperandrogenism (hirsutism score based on hirsutism score greater than 7 or obvious acne) and/or an elevated plasma testosterone (testosterone >2. 0 nmol/l); (iii) having an interval between menstrual periods >35 days and /or amenorrhea, defined as the absence of vaginal bleeding for at least 6 months (i.e.,199 days). Once women agreed to participate and, following consent into the study, interview appointments were arranged.

### Participants

Out of the 30 women who invited to participate in the study, 10 declined (reasons unknown). The final sample consisted of 20 patients with PCOS. The characteristics of the sample are detailed in Table 1. The age range of participants was 21–34 years. Most participants were educated to diploma level but were unemployed.

### Interview schedule

The study utilized a semi-structured interview that broadly aimed to explore the impact PCOS had, or was having, on women’s lives. Interviews started with general questions such as “tell me about your experiences and perceptions about PCOS”.

### Data collection

Interviews were conducted by FB and were audio recorded. Interview length ranged from 20 min to 1:29 h (mean: 42 min). Interviews were transcribed verbatim by FB to playscript standard.

### Data analysis

A thematic analysis was conducted on the interview data, following the approach detailed by Braun and Clarke (2006) [8]. Thematic analysis is a flexible research tool, which can support the generations of detailed analysis without being restricted by a particular theoretical (i.e., epistemological) position. It aims to identify, analyse, and report patterns (themes) within data. Following multiple readings of the interviews, analysis involved line-by-line coding, whereby descriptive codes were assigned to sections of text that had distinct meaning (e.g., feeling pressured. Next, similar codes were grouped to create the categories (e.g., an unsexualised self: loss, change and pain,being pained) subsequently categories were condensed to form themes (e.g., sexual - physical problem). Data entry and analysis were supported by MAXQDA software available at http://www.maxqda.com. MAXQDA facilitates data management, the assignment of labels, codes and themes to text fragments and the generation of thematic matrices containing these elements. In the past, MAXQDA software has been successfully used in similar studies [9].

The primary analysis was conducted by FB. Emerging analytic claims were discussed with the study team, to test the credibility of analysis based on the raw data. Credibility was further enhanced through member checks. Some studyarticipants (*n* = 20) were contacted post-analysis, were given a full transcript of their coded interviews, and asked to indicate whether coding was appropriate for their experiences. Agreement between researcher and participant was above 95 %. Maximum variation of sampling as well as prolonged engagement increased the credibility of data.

There are numerous approaches for analyzing qualitative data. Content analysis is a method of analyzing written, verbal or visual communication messages [10]. An advantage of the method is that large volumes of textual data and different textual sources can be dealt with and used in corroborating evidence. A qualitative content analysis method was used to reach the objective of the study using conventional method. In conventional content analysis, coding categories are derived directly from the text data [11]. Therefore, we selected qualitative content analysis study for present research.

## Results

The analysis suggests that PCOS is a physical/sexual, psychological and social syndrome. Findings are presented as three themes; (i) sexual - physical problems; (ii) exposure and invasion; and (iii) diminished self and diminished life.

### Sexual - physical problems

This theme is presented with two sub-themes, which capture the effects that PCOS has on women’s sexual life (an unsexualised self) and on their body (being pained).

#### An unsexualised self: loss, change and pain

Many participants talked about the negative impact of PCOS and its treatment on their marital relationships, particularly in the realm of sexual desirability and exposure of oneself. They talked about psychological and physical barriers to a fulfilling sexual relationship. For example, some women’s desire for sex was diminished by feelings of shame about the symptoms of PCOS (“with too much hair on my body I don’t feel like having any sexual intercourse.” [P aged 25]). orr the pain they experienced during sex (“I have zero sexual desire. I prefer to finish soon for the severe pain resulted from the drugs I took for ovulation” [P aged 30]).

Infertility associated with the syndrome also had a profound effect on couple relationships. Women talked about how the nature of sexual relations altered in an ‘infertile context’, profoundly diminishing desire and enjoyment of sex: “Our sex does not give results, and we fail to have a baby. After this, I don’t have any desire, I hate sex, I have no sexual satisfaction at all” [P aged 23]. Moreover, often in infertility treatment, a specific timetable is recommended for intercourse, and these schedules impacted the naturalness of sexual activity between couples. However, abiding to schedules increases the possibility of becoming pregnant, and some women considered the trade-off between sexual enjoyment and being ‘signed off’ scheduled sex: “Sometimes, I am tired. I’d like to rest at night… then I say No! It’s better to have a baby and get released! I wish to have a baby as soon as possible and be released from timetabled sex!” [P aged 28].

#### Being pained

Many participants talked about the physical side effects of PCOS and its treatment on their general health. A dominant concern was the pain associated with managing and treating excessive hair re-growth and emerging acne. Women engaged with multiple practices to beautify or feminize themselves, despite discomfort and pain: “I did not like the excessive hair on my breasts. I always removed it with tweezers… this is very painful” [P aged 24]; “After removal of hairs, thick hairs grow back, itching and painful” [P aged 28].

Other physical complications associated with treatments included pain related to diagnositic examinations (“My first internal examination was because of severe pain and bleeding, because of that, I am afraid of it” [P aged 28]), or effects of particular drugs: (“When taking this medications, I was always hot, and had hot flushes. I felt pain in my lower abdomen” [Paged 27]; “Taking the pills, I was too anorexic with very hot flushes” [P aged 24].

### Exposure and invasion: the rejecting and invading social world

This theme captures the aspects of women’s experiences which were heavily contextualized in their immediate social worlds where they felt simultaneously scrutinized and rejected, leading to a desire to conceal themselves and avoid others (Concealing and Avoiding), and the positioning by others as public property, leading to feelings of intrusion (Public property: public scrutiny).

#### Concealing and avoiding

Participants described difficult experiences whereby the reactions of others led them to feeling marginalized: “Everyone pays attention to me. So, I do not go to their home. I do not feel comfortable there. When they tell me that I am fat …I resent it, and am really affected by it” [P aged 27]. Some participants refused to participate in parties and ceremonies (“Now, I don’t go out much. Unlike before, I’m not happy going to wedding parties” [P aged 22]; “I am disturbed by the menstrual irregularities because if I decided to go a ceremony or wedding, I might get a heavy period” [P aged 25]); and one woman shut out the world for a substantial period of time: “For two years, I did not even turn on the TV.” [P aged 27]. Thus, having PCOS had a very real impact on women’s felt freedom of movement, and their perceived position in their own social worlds. Participants also talked of concealing their symptoms from others, to reduce likely negative reactions or intrusions: “If I go to my mother’s house, I always cover my cheek to conceal acne” [P aged 23]. When asked ‘what has been the impact of your hirsutism on your life’, another woman stated: “I have too much hair and am really preoccupied about it. I don’t like others to see me with too much hair. I wear a scarf to conceal the excessive hair on my face. If people see me with this hair, they tell me that my face looks like a man’s and we cannot tell you from your husband! I try to wear long hair at home to cover my face from my husband” [P aged 23].

Here, the participant discloses a preoccupation with hair growth and a felt imperative to conceal it form others, including her partner. The implication is that the hair growth could not be, or would not be well tolerated by others (who may or may not know about the PCOS diagnosis) and that it is a woman’s responsibility to ‘protect’ others from seeing the reality of hair growth with PCOS. The woman is also motivated to be protective of herself, given the intolerance she may experience in her social world. Thus, women with PCOS are highly exposed and highly vulnerable given the visibility of their symptoms. Their options in this context are ones of avoidance, concealment and protection.

#### Public property: public scrutiny

Most participants spoke about the reaction of people in their immediate social worlds to the symptoms of PCOS. One of the issues expressed by participants was the extent of false beliefs about PCOSwhich led to the exaggeration of symptoms and patient’s discomfort: “If I don’t remove these hairs, everyone tells me: woo! You have too much hair growth. These are a sign of your infertility. It is a mental burden” [P aged 23]. “My friend told me that, since long time has passed pastfrom your fertility course there may be no solution” [P aged 34].

Some participants were affected by recommendations of others during the process of treatment: “For my menstrual cycle, I should take a progesterone shot. Some told me those injections are not good and that they mess with your fertility. They said that you should have a spontaneous menstrual cycle not one caused by injection. Sometimes, I feared the side effects of those shots and so I stopped taking them. Therefore, I did not get my period and then this really affected my mentally” [P aged 25].

Across these extracts, people are reported as assuming the right medicalise the woman’s body, claiming ways in which her body was faulty or broken (“too much hard growth”; “there is no solutions”). Others’ apparently well-meaning advice sought to re-naturalise the woman’s body (“you should have a spontaneous menstrual syccle”). These claims and pseudo-diagnoses of what the woman’s body should be like appeared to upset women, and presented an additional burden to that of managing the syndrome privately.

In particular, pregnancy vs. infertility appeared to be available for public scrutiny. With prolonged treatment of PCOS, the curiosity and intrusion of others increased and caused participants discomfort@ “They are always asking me why I did not plan to get pregnant? Why is that ? What seems to be the problem? These are always their first questions” [P aged 30].

Some participants felt that they were stigmatized in the community due to infertility: “I lived in a village and I had to hide my visit to a gynecologist since people did not have a good view of that. Because, in the village everyone knows each other - I was single and my visit to a gynecologist surprised them, and they asked… ‘what is her problem that forced her to visit a gynecologist’” [P aged 21].

“One told me: when I see that you are infertile, I am worried….so I’m going to have children as soon as possible!”[P aged 27].

Taunting was another experience endured by women with PCOS, typically from men who challenged both femininity as well as the truth of their experiences: “My husband always says: Oh! You have too much hair! You are just like a real man” [P aged 23].

“I have irregular menstruation. I can’t pray some days. Some men surprisingly used to ask me: Are you always on your period?! They think that I evade praying and make up excuses” [P aged 28].

Thus, across a range of aspects of PCOS, women reported feeling invaded by a public gaze, which both sought to act as pseudo-medical informants, as well constant challenges to the normality of their bodies, and their womanhood.

### Diminished self and diminished life

Across the transcripts, women talked in ways which reflected their sense of being diminished by their experience of POCS, but largely in terms of the reactions of others, rather than coping with the syndrome in and of itself. This set of sub-themes captures the psychological and emotional impact of POCS: (i) Infertile as inferior and (ii) Exhausted mind and body.

#### Infertile as inferior

Feeling inferior, and less of a woman, was talked about often, and mostly in relation to being infertile – and how people in women’s social worlds assigned meaning to that and therefore shaped women’s own understanding and experience. For example: “since their pregnancy, they always show off and and call me infertile. I always feel inferior” [P aged 29]; “Even at a wedding or a party, I’m ashamed to talk with someone who got married after me and has a baby” [P aged 27].

The imperative to become pregnant is profound among Iranian women and being infertile was a very difficult reality for study participants to tolerate. “It is very bad situation. My life is so hard since we stopped thinking of having a baby. It’s awful.” [P aged 32]. Feelings of being less than a woman, or of being slow in assumed progress of a woman’s life, were prominent and sometimes extreme: “When I see a pregnant woman or those with kids, I envy so much that I want to kill them” [P aged 32].

“I’m sometimes so ashamed of my husband because of the excessive hair. I wish I never had him.” [P aged 34].

Being infertile, or yet to become pregnant, was central to womanhood for the study participants. They reported feelings of being consumed with thoughts of becoming pregnant, of extreme jealously of others with children and deep loss following decisions to remain childless.

#### Exhausted mind and body

For many, the mental and physical tiredness and weariness of coping with PCOS permeated their lives substantially, severely impacting their capacity for normal functioning. For example, women talked about the physical drain of PCOS (“Sometimes, I get so tired and tell God: please kill me or cure me” [P aged 27]) and how their diminished resources meant they had low tolerance for everyday activities: “I am not interested in daily tasks. After two weeks, I stopped using the vacuum cleaner to clean our home because I could not tolerate the noise of the vacuum cleaner” [P aged 32]; “I am not patient now, I can’t stand anything” [P aged 22]. Feelings of depression and hopelessness were also cited, especially after prolonged treatment, and showing how women shifted from experiences of determination and coping to one of resignation: “Before, I did not think my problem was so acute and difficult to treat. I thought PCOS was no big deal. I can treat it through taking some medications or injections immediately. But, now I am more hopeless than ever. I think there is no solution” [P aged 32]. Looking after themselves, or investing in themselves, seemed difficult for some women. For example, one woman stated: “now, maybe I go to beauty salon twice a month under the pressure of my husband. My husband always tells me: “you were better before, you are like an old woman now” [P aged 25]. Here, not only is the woman coping with the draining nature of her condition, but she is still called upon to invest effort in her presentation to others.

Engagement related to the disease and its complications was another problem in these participants who reported feeling constantly immersed in thoughts about their illness, the effects of treatment, and what the future may hold for them: “I always told myself: if I become pregnant in this condition, those drugs may have negative effects on my baby” [P aged 32].

“I feel my life is useless because I don’t have any baby. On the other side, as the age increases, the treatment becomes more complicated.” [P aged 23].

## Discussion

Being diagnosed with, and treated for, PCOS can have a profound effect on women’s lives. This study reports the outcomes of an interview based study with Iranian women diagnosed with the syndrome. Iranian women may face particular experiences related to PCOS given particular social and cultural imperatives. Our study reports three themes which capture the multiple ways in which PCOS affects the women’s quality of life and how this is largely mediated by their sense of self and shaped by the reactions of significant others in their social world.

Many symptoms of PCOS are painful, unpleasant and unpredictable which are associated with features that are culturally “non-feminine” and considered undesirable. Previous findings indicate that women with PCOS feel ‘freakish’, ‘abnormal’, and not ‘proper’ women [11, 12], reflecting the perceived inability to conform to the socially prescribed characteristics of femininity. Teede et al. [13] stated that PCOS is a frustrating experience for women and have negative effects on their mental state and subsequently the patient’sHRQOL [12, 13]. In Himelein and Thatcher’s study, patients with PCOS often experienced depression, isolation, anxiety and frustration [13, 14].

PCOS is often diagnosed at the time in life when finding a sexual partner, beginning sexual activity and marriage is important. Therefore, it is thought that the femininity and psychosexual issues related to PCOS can cause substantial distress for these women [14, 15]. PCOS related changes in appearance may contribute negatively to sexual satisfaction and sexual self-worth, possibly by affecting self-esteem and female identity. Studies show that diseases such as PCOS cause loss of body control and a sense of uselessness leading to a negative body perception [15, 16]. Women with PCOS reported that they do not feel their body is sexually appealing. Our results are in line with the findings of de Niet et al. [17] showing that PCOS symptoms might be negatively associated with self-esteem, body satisfaction, and/or fear of negative appearance evaluation [16, 17]. It has been shown that not only the visible features of PCOS, such as higher body weight and excessive growth of bodily hair, were related to increased experience of fear of what other people thought about patients’ appearance, but also the absence of their cycle (amenorrhea) was negatively associated with fear of appearance evaluation [16, 17].

The importance of menstrual irregularities for Iranian PCOS women has been demonstrated in an earlier study which found that menstrual problems were the greatest concern reported by women [17, 18]. Menstrual irregularities can have important social consequences, especially in Muslim settings. For example, the tenets of Islam decree that menstruating women cannot pray [6]. If a woman is visibly not engaging in prayer for more than the expected 4 or 5 days per month, her whole household and social entourage are likely to be aware that she is experiencing menstrual irregularities [18, 19]. Therefore, prolonged bleeding disrupts household patterns in such a way hat family and community members may become aware of a woman’s situation if her period persists for more than the expected number of days. Similarly, amenorrhea may signal menstrual disruption to family members who may notice that the expected 4–5-day monthly break in the woman’s religious or household duties. Menstrual irregularities also may have consequences on women’s intimate relations and other aspects of their reproductive and general health. For example, Islamic texts forbid men from having sex with menstruating women. The findings from the present study are in general agreement with an earlier study reporting that Muslim women with PCOS rated menstrual irregularity and infertility as their greatest concerns [19, 20].

It should be noted that the extent of infertility on HRQoL varies according to socio-cultural factors, traditions, and religious beliefs. It is possible that women from different ethnic and cultural backgrounds have a different perception of their body and sexual functioning, offering a potential explanation for variation in experience. Iranian women who are infertile are under social pressure to have children; therefore, infertility may have more impact on these women compared with those from a Western culture. Our study was conducted in a developing country where the majority of people were Muslims. According to the Islamic beliefs, marriage is most of all a way to procreate and to ensure the formation of a family [20, 21]. Given this, infertile Iranian women are under high pressure to procreate, and infertility might affect them more compared with women from western cultures. The findings form of the current study, are in general agreement with an earlier study by Winkvist and Akhtar [21, 22]. Many Islamic Pakistani women felt strongly that their childbearing ability influences the way people treat them; they are more respected when they have children. Without children, they do not feel like a real woman [21–23]. “Not having baby” is a substantial psychological burden that is often associated with divorce and loss of social prestige because “mother” is considered an important part in maintaining female identity [23]. As a result ‘failure’ in tasks such as reproduction decreases women’s self-confidence and self-esteem. Infertility is considered a physical disability and these women feel that they have a physical defect. Therefore, it is conceivable that infertility (e.g., chronic disease) is associated with an impaired body image. Moreover, it was recently reported that infertile PCOS women had significantly higher depression scores and greater body dissatisfaction compared with the women with infertility from causes other than PCOS, which would also support the contribution of PCOS factors other than infertility [13, 14].

In the present study, inappropriate reactions in women’s social worlds, and reduced, interpersonal interactions were reported social consequencesof the syndrome. Ekback and colleagues [24] in their qualitative study showed that hirsutism causes severe psychological burden that negatively affected patients’ HRQoL and social interactions [24].

Our findings also revealed that the stigmatizing nature of PCOS in Iranian communities is shaped by persistent cultural beliefs about…?; thus, some participants hide the illness from others.

Concealment may have a negative impact on patients’ self-respect and may affect their relationships with others and threaten their social life.

Healthcare providers who work with women diagnosed with PCOS in Iran should be aware that women’s HRQoL with PCOS is affected by cultural and religious issues. We hope that these steps will lead to improved HRQOL in Iranian patients with PCOS and that problems, limitations, and stresses in their daily lives can be alleviated. Although this study was strong in many ways, it was not without limitations. Iranian unmarried women generally avoid visiting the doctor for cultural issues. For the same reason as well as illegitimacy of sexual relation and having prior to marriage children, the population of present study compromised married women with PCOS. Therefore, the generalisability of the findings to unmarried women with PCOS may be limited.

## Conclusion

The results of this study suggest that PCOS is a physical - sexual, psychological and social syndrome; therefore, it is necessary to take a more holistic approach to patient care beyond treating physical symptoms. Reproductive health specialists, more than other healthcare professionals, can address poor HRQOL in PCOS patients. Using interviews and open ended questions provide an opportunity for participants to express their concerns about quality of life issues. Future research should seek to better understand quality of life as it relates to different socioeconomic backgrounds and ethnic and racial characteristics in Iran. These studies provide an opportunity to help design culturally sensitive interventions in Iranian society to further improve the quality of life of PCOS patients.

## References

[1] Ehrmann DA (2005). Polycystic ovary syndrome. N Engl J Med.

[2] Hahn S, Janssen OE, Tan S, Pleger K, Mann K, Schedlowski M (2005). Clinical and psychological correlates of quality-of-life in polycystic ovary syndrome. Euro J Endocrinol..

[3] Bazarganipour F, Ziaei S, Montazeri A, Foroozanfard F, Kazemnejad A, Faghihzadeh S. Psychological investigation in patients with polycystic ovary syndrome. Health Qual Life Outcomes. 2013;11(1):141–150 10.1186/1477-7525-11-141 PMC3751454 23947827

[4] Mansson M, Holte J, Landin-Wilhemsen K, Dahlgren E, Johansson A, Landen M (2008). Women with polycystic ovary syndrome are often depressed or anxious: a case control study. Psychoneuroendocrinology.

[5] López EDS, Eng E, Randall-David E, Robinson N (2005). Quality of life concerns of African American breast cancer survivors within rural North Carolina: blending the techniques of photo -voice and grounded theory. Qual Health Res.

[6] Omran AR (1992). Family planning in the legacy of Islam.

[7] Gottlieb A (1982). Sex, fertility and menstruation among the Beng of the lvory coast: a symbolic analysis. Africa (Lond).

[8] Braun V, Clarke V (2006). Using thematic analysis in psychology. Qual Res Psychol.

[9] Kronenwetter C, Weidner G, Pettengill E, Marlin R, Crutchfield L, McCormac P (2005). A qualitative analysis of interviews of men with early stage prostate cancer: the Prostate Cancer Lifestyle Trial. Cancer Nurs.

[10] Cole FL (1988). Content analysis: process and application. Clin Nurse Spec.

[11] Hsieh HF, Shannon SE (2005). Three approaches to qualitative content analysis. Qual Health Res.

[12] Kitzinger C, Willmott J (2002). The thief of womanhood’: women’s experience of polycystic ovarian syndrome. Soc Sci Med.

[13] Teede H, Deeks A, Moran L (2010). Peovielwycystic ovary syndrome: a complex condition with psychological, reproductive and metabolic manifestations that impacts on health across the lifespan. BMC Med.

[14] Himelein MJ, Thatcher SS (2006). Polycystic ovary syndrome and mental health: a review. Obstet Gynecol Surv.

[15] Eggers S, Kirchengast S (2001). The polycystic ovary syndrome-a medical condition but also an important psychosocial problem. Coll Anthropol.

[16] Smolak L, Levine MP, Thompson JK (2001). The use of the sociocultural attitudes toward appearance questionnaire with middle school boys and girls. Int J Eat Disord.

[17] de Niet JE, de Koning CM, Pastoor H, Duivenvoorden HJ, Valkenburg O, Ramakers MJ (2010). Psychological well-being and sexarche in women with polycystic ovary syndrome. Hum Reprod.

[18] Bazarganipour F, Ziaei S, Montazeri A, Frozanfard F, Faghihzadeh S (2013). Health related quality of life and its relationship with clinical symptoms among Iranian patients with polycystic ovarian syndrome. Iran J Reprod Med.

[19] Gottlieb A (1982). Sex, fertility and menstruation among the Beng of the Ivory Coast: a symbolic analysis. Africa (Lond).

[20] Schmid J, Kirchengast S, Vytiska-Binstorfer E, Huber J (2004). Infertility caused by PCOS—health-related quality of life among Austrian and Moslem immigrant women in Austria. Hum Reprod.

[21] Fido A, Zahid MA (2004). Coping with infertility among Kuwaiti women: cultural perspectives. Int J Soc Psychiatry.

[22] Winkvist A, Akhtar HZ (2000). God should give daughters to rich families only: attitudes toward childbearing among lowincome women in Punjab, Pakistan. Soc Sci Med.

[23] Penn R, Lambert P (2002). Attitudes toward ideal family size of different ethnic/nationality groups in Great Britain, France and Germany. Popul Trends.

[24] Ekback M, Wijma K, Benzein E (2009). It is always on mind: women’s experiences of their bodies when living with hirsutim. Health Care Women Int.

